# Editorial: Molecular mechanisms in psychiatry 2023: addictive disorders

**DOI:** 10.3389/fpsyt.2025.1568150

**Published:** 2025-02-17

**Authors:** Lucía Fernández-López, Pilar Almela, Maria Falcón, Javier Navarro-Zaragoza

**Affiliations:** ^1^ Department of Pharmacology, University of Murcia, Murcia, Spain; ^2^ Department of Health and Social Sciences, University of Murcia, Murcia, Spain

**Keywords:** addiction, alcohol, nicotine, serotonin receptor (HTR2A) gene, mobile phone (or smartphone) use, epigenetics, inflammatory diseases

**Figure 1:**
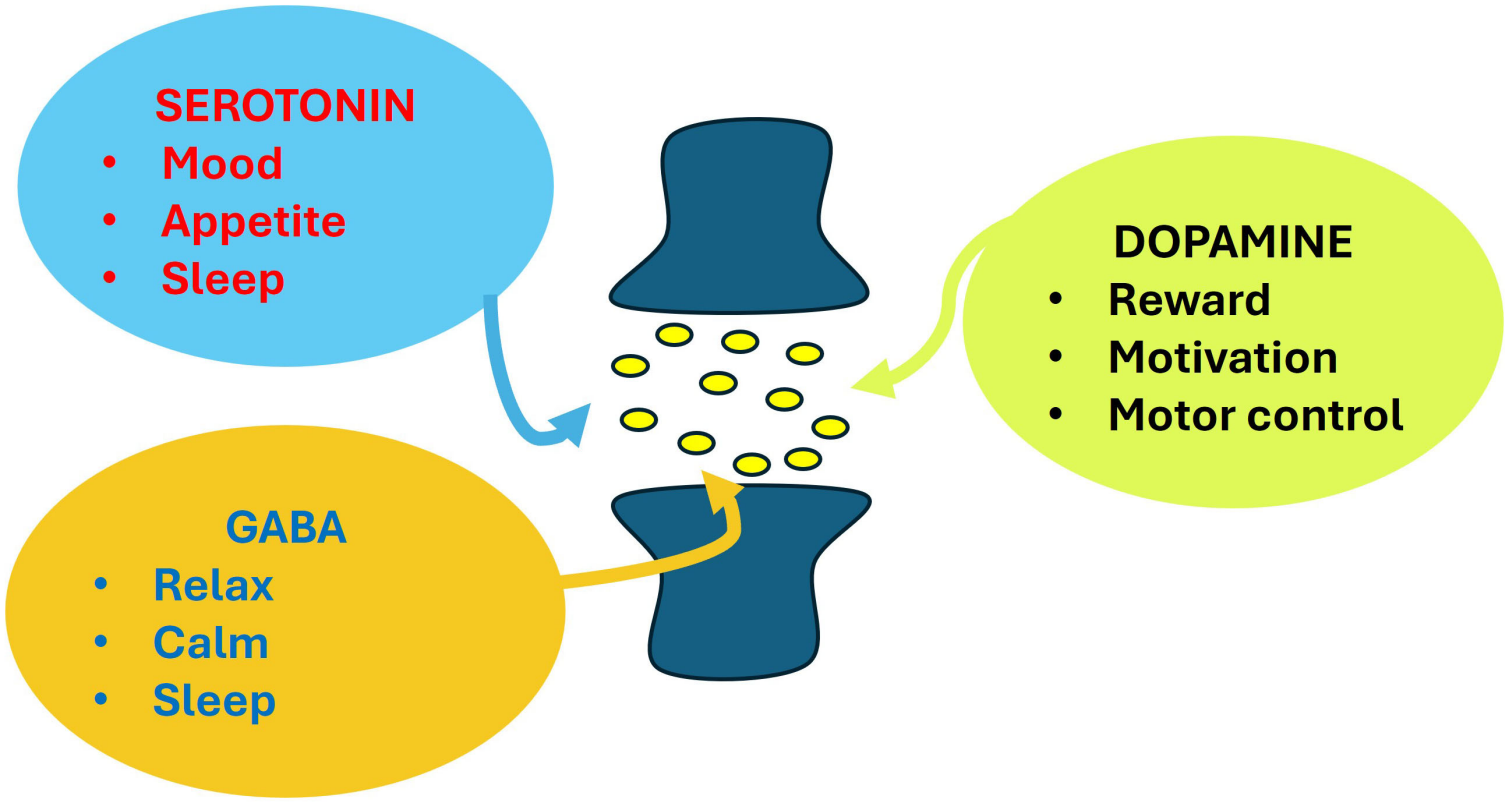
Different effects of neurotransmitters such as dopamine, GABA and serotonin.

Addiction disorders involve complex molecular mechanisms that affect neurotransmission systems in the brain, primarily in the reward system, which includes key structures such as the nucleus accumbens, prefrontal cortex, and ventral tegmental area (VTA). Dopamine is essential for the sensation of pleasure and positive reinforcement. Drugs of abuse increase dopamine release in the nucleus accumbens, reinforcing addictive behaviour (1). Other involved neurotransmitters are glutamate, GABA (gamma-aminobutyric acid) or serotonin (5-HT). Serotonin is a neurotransmitter that plays a crucial role in regulating various physiological and psychological functions in the body. It is commonly known as the “happiness hormone” because its release is correlated with well-being and emotional balance (**Figure 1**). It is involved in regulating mood, sleep, appetite, digestion, social behaviour and impulse control, which are altered in addiction (2). A very interesting manuscript about the serotonin receptor 2A has been included in this Research Topic. Drug dependence produces important changes in the organism, both cellular and molecular level, resulting in sensitization, tolerance, dependence, and there always be the threat of relapse.

Chronic drug exposure can also alter gene expression (3) through epigenetic changes which are mechanisms that regulate gene expression without altering the DNA sequence. These factors can turn genes on or off in response to environmental influences, such as, exposure to drugs. Epigenetic factors alter DNA methylation and produce histone modifications that affect gene expression related to neuronal plasticity which affects decision-making and self-control (4).

Six articles have participated in this Research Topic providing relevant information regarding addictive disorders. Three of these articles deal with genetic polymorphisms. Dai et al. have studied the association between rs6313 (T102C) polymorphism in the serotonin 2A receptor (5-HT2A) gene and Internet Addiction Disorder (IAD). Besides, Yang et al. have shown how specific genetic variations influence impulsivity in individuals with Alcohol Use Disorder (AUD). Furthermore, the study focuses on two polymorphisms: BDNF rs6265 (Val66Met) which affects the production of Brain-Derived Neurotrophic Factor, that is crucial for neuronal survival and plasticity, and FGF21 rs11665896 which influences the production of Fibroblast Growth Factor 21, involved in metabolic regulation. In addition, Eskandarion et al. have established a relationship between genetic variations in the Glutathione S-transferase (GST) genes and addiction to opioids and methamphetamine. Opioid abuse is a concerning problem in the USA nowadays and to explore the relationship between genetic differences and opioid addiction could be of great interest.

Two articles of this Research Topic discuss the consequences of addictive processes in physiological alterations and the subsequent development of pathologies. In fact, Kridin et al. have studied the association between nicotine dependence and the risk of developing chronic, non-communicable inflammatory diseases (CIDs). The study emphasizes the importance of preventive measures targeting nicotine addiction to reduce the global burden of CIDs. Previous studies have investigated the influence of drug use on the development of inflammatory diseases, finding that the brain exhibits marked oxidative stress and neuroinflammation following chronic drug use (5). Furthermore, Mei et al. have focused on the impact of mobile phone addiction on the circadian rhythms of saliva microbiota. In recent years, the relationship between microbiota alterations and different mental illnesses has been reported (6).

Finally, the article “*A critical scientific evaluation of a purportedly negative data report – response to Seneviratne et al., 2022*”, is a critical evaluation of a negative data report published by Seneviratne and colleagues in 2022. The authors, Johnson et al., have discussed the importance of replicability in scientific research and highlight how errors in negative data sets can arise from methodological, statistical or conceptual flaws, as well as flawed peer review processes. Furthermore, they discuss how the publication of false negative data can negatively impact scientific progress, in this case, in the context of the use of ondansetron for the treatment of alcohol use disorder. In this regard, research indicates that the pleasurable effects of alcohol are related to the activation of serotonin 5-HT3 receptors, which trigger the release of dopamine in the mesolimbic system of the brain, thereby increasing the likelihood of alcohol craving and misuse. Therefore, ondansetron, by inhibiting the activation of 5-HT3 receptors, may help to decrease alcohol-induced dopamine release, resulting in a decreased sense of reward and, in turn, a reduction in craving and alcohol consumption (7).

Therefore, all these studies focus on a wide range of molecular mechanisms that are responsible for the different changes observed in addictive disorders and delve into the physiological alterations and genetic polymorphisms involved in addictive disorders. In summary, this Research Topic is a good overview of the latest advances in research aimed at understanding the causes and effects of drug addiction.
